# The Sum of Two Halves May Be Different from the Whole—Effects of Splitting Sequencing Samples Across Lanes

**DOI:** 10.3390/genes13122265

**Published:** 2022-12-01

**Authors:** Eleanor C. Williams, Ruben Chazarra-Gil, Arash Shahsavari, Irina Mohorianu

**Affiliations:** 1 Wellcome-MRC Cambridge Stem Cell Institute, University of Cambridge, Cambridge CB2 0AW, UK; 2 Life Sciences-Transcriptomics and Functional Genomics Lab, Barcelona Supercomputing Center (BSC-CNS), 08034 Barcelona, Spain

**Keywords:** mRNAseq, ChIPseq, smartSeq, 10×, sample splitting, differential expression, enrichment analysis, cell type calling

## Abstract

The advances in high-throughput sequencing (HTS) have enabled the characterisation of biological processes at an unprecedented level of detail; most hypotheses in molecular biology rely on analyses of HTS data. However, achieving increased robustness and reproducibility of results remains a main challenge. Although variability in results may be introduced at various stages, e.g., alignment, summarisation or detection of differential expression, one source of variability was systematically omitted: the sequencing design, which propagates through analyses and may introduce an additional layer of technical variation. We illustrate qualitative and quantitative differences arising from splitting samples across lanes on bulk and single-cell sequencing. For bulk mRNAseq data, we focus on differential expression and enrichment analyses; for bulk ChIPseq data, we investigate the effect on peak calling and the peaks’ properties. At the single-cell level, we concentrate on identifying cell subpopulations. We rely on markers used for assigning cell identities; both smartSeq and 10× data are presented. The observed reduction in the number of unique sequenced fragments limits the level of detail on which the different prediction approaches depend. Furthermore, the sequencing stochasticity adds in a weighting bias corroborated with variable sequencing depths and (yet unexplained) sequencing bias. Subsequently, we observe an overall reduction in sequencing complexity and a distortion in the biological signal across technologies, experimental contexts, organisms and tissues.

## 1. Introduction

The recent developments and improvements in high-throughput sequencing (HTS) technologies have facilitated increasingly complex transcriptome/genome-wide analyses [1], enhancing both the qualitative annotation of genomes [2,3,4] and their quantitative, functional characterisation through differential expression studies [5,6]. The diversification of methods specialised to a wide range of perspectives on DNA/RNA biology [7] was complemented by studies at the single-cell level [8]. Advances were observed across all aspects of the sequencing workflow [9], complemented by an increasing amount of resulting data. This created another challenge: producing robust and reproducible results and simultaneously keeping up with the increasing intricacy of experiments [10].

The variability of sequencing output, which propagates through to quantification and other downstream exploration, poses one of the main challenges in bioinformatics analyses since it implies the disentangling of relevant from irrelevant sources of variation. While the biologically relevant quantities are context-dependent [11], an essential distinction exists between variability due to biological processes and variability due to measurement error or inaccuracy [12,13]. The former is generally specific and well defined in relation to a condition; even when it is perturbed by noise, an underlying pattern of expression may emerge [14]. Technical variability encompasses measurement error [15], sequencing bias [16,17], and variability due to missing data [18]. For the latter, the assessment of technical variation can be hindered by the lack of a ground truth.

Several studies have proposed approaches to identify and characterise the sources of variability in HTS experiments, focusing on several aspects of signal distribution, which can affect the accuracy of the downstream analyses and interpretations and jeopardise the reproducibility of the results [11,19]. These included both the analysis of noise [14,20] and the downstream components of the analyses, such as the batch/background effect [21], alignment approaches [22], processing pipelines [5], normalisation methods [23] and differential expression thresholds [24,25]. To model the intrinsic biological variability, the number of replicates in the context of experimental design was optimised using power calculations [11,26], designed to provide a robust estimation of differences in expression. These approaches rely on simulations on the number of expressed genes, as well as on mean-dispersion estimates and dropouts after applying frequency and outlier filtering; traditional approaches do not take into account elements of sequencing design including across-lane sample splitting. In general, the impact of library construction and flow cell and lane characteristics on downstream analysis has not been studied in detail.

Sources of technical variability for RNAseq experiments span from the combinatorial numbers of highly variable isoforms to the handling of ambiguous or multi-mapped reads [1]. For ChIP datasets, the ability to address specific biological questions can be significantly impacted by antibody efficiency and specificity [27], as peak distributions are a direct consequence of affinity, over-crosslinking, DNA fragmentation and PCR amplification; for such samples, users are faced with a trade-off between the number of usable reads (sensitivity of peak detection) and the proportion of false positives derived from multi-mapped reads [28]. Low-quality replicates can also generate bottlenecks when used in conjunction with good samples, as true peaks missing from poor-quality replicates may be marked as non-reproducible, thus creating false negatives [27]. Single-cell experiments share some of the drawbacks of bulk ones; in addition, the exponential increase in the number of cells profiled per study, coupled with the shallower sequencing depth, redefined some of the known difficulties, such as the characterisation of noise [20,29].

Traditional batch effects stem from various sources, e.g., processing differences due to human variability, differences in achieved sequencing depth, or amplification/sequencing bias across runs. In addition, the variation in mRNA capture efficiency, the strand specificity for overlapping genes or the fluctuations in transcript coverage have all been thoroughly studied [30] and quantified [31]. These batch effects were shown to impact downstream analyses and induce effects confounded with biological signals; these biases cannot be mitigated using standard normalisation methods and consequently result in a loss of statistical power or misleading results [32]. Several approaches were developed and benchmarked [21,33] to salvage datasets with evident batch effects, including ComBat-seq [34], which is based on negative binomial regression, and POIBM [35], which infers virtual reference samples for bulk RNA-seq data. Methods developed for single-cell RNA-seq data include mnnCorrect [36], based on mutual nearest neighbour detection, CarDEC [37], a deep learning approach, and scBatch [38], which finds a linear transformation retaining the advantages of QuantNorm [39]. The quantification of gene expression summarises a mixture of batch effects; the task of regressing out these effects, while preserving true biological variation, has been acknowledged as difficult.

Here, we investigate a controllable source of variation: the effect of across-lane sample splitting, at the sequencing stage, on downstream analyses for bulk and single cell data; the sampling approach is modelled on observed sequencing outputs (bulk mRNAseq data). To infer the effect on other types of sequencing data, we study the differences between ground truth and split-samples (simulating across-lane splitting), with various parameters controlling the number of splits and proportion of reads produced on each lane. We focus on standard analyses, i.e., the identification of differentially expressed (DE) genes or ChIP peaks for bulk analyses; for single-cell analyses, we concentrate on the allocation of cells to clusters (viewed as proxies for cell types) and comment on the observed variability in biological interpretations.

## 2. Materials and Methods

### 2.1. Materials

The motivation for the subsampling strategy used throughout the manuscript is derived from a *D. melanogaster* mRNAseq dataset (GSE85806) for which 3 samples were sequenced and split across 2 lanes (GSM2284703, GSM2284704 (2RA3), GSM2284705, GSM2284706 (2RH2), GSM2284707, and GSM2284708 (26RH3)). To understand and highlight the consequences of this choice in sequencing design, we compared the resulting expression levels to the corresponding full samples, GSE55839 (GSM1346985 (2RH2), GSM1346996 (2RA3), and GSM1347001 (26RH3)) [40].

To illustrate the split effects and their link to the biological interpretation, we use bulk and single-cell mRNA data and bulk ChIPseq data. For the former (bulk mRNAseq), we used the Yang et al., 2019 dataset [41], focusing on the 0 h and 12 h samples (GSE117896, comprising SRR7624365, SRR7624366 (biological replicates for 0 h), SRR7624371 and SRR7624372 (biological replicates for 12 h)). The bulk ChIPseq analysis was performed on H3K4me3 and H3K27ac samples, using 0 h and 12 h samples for each (SRR7624381, SRR7624384, SRR7624389, SRR7624392).

To exemplify the effect on plate-based scRNA-seq platforms (smartSeq), we used the Cuomo et al., 2020 dataset [42]. We selected data from 6 donors on 4 time points. On this input, six experimental study cases (Table 1) were designed to illustrate the effect of the different covariates, i.e., the donor, the specific time-point and cell type (resulting cluster) identities. To investigate the effects of lane-splitting on 10× Genomics scRNA-seq data, we used an in vivo dataset of human hematopoietic stem and progenitor cells from spleen, bone marrow, and peripheral blood [43]; the data are available via BioStudies accession number S-SUBS4 (donor SAMEA6646089).

### 2.2. Methods

**[Splitting strategy—sequenced samples]** For the 3 *D. melanogaster* samples (2RA3, 2RH2, 26RH3) for which whole-lane and split-lane sequencing was available, we followed the standard mRNAseq quantification procedure; the split samples were merged without any additional pre-processing (merged-samples). Whole, split and merged samples were aligned to the *D. melanogaster* r6.41 genome [44] using STAR 2.7.0a [45] with default parameters. Next, the expression was quantified using featureCounts 2.0.0 [46] and summarised into count matrices. For each BAM, a bigwig was produced using bamCoverage, and individual transcript coverage was identified using pyBigWig from deeptools [47]. In addition, for all settings, we determined the number of non-redundant (unique) and redundant (all) reads and evaluated the number of fragments present exclusively in the one setting. We also calculated the ratio between the abundance of a read (its redundancy) in the whole vs. split sample, with an expected value equal to the ratio of sequencing depths.

**[Splitting strategy—simulated data]** The splitting strategy for the simulation study is consistent across all datasets. The splitting is performed per sample. For each dataset, on the ground truth (GT), i.e., the original sample, and on the simulated, split (S) samples, the same pipelines (for the alignment, quantification and identification of differentially expressed entries), with identical, default parameters are applied to ensure an unbiased comparison between results obtained on the GT and S samples, respectively.

Let *k* be the number of splits for a sample and *n* be the number of iterations (each split sample is generated from the original GT sample). The steps for generating an S sample are: (1) subsampling reads without replacement from the GT sample to $\frac{100}{k}\%$ of GT in *n* iterations; (2) subsequently, the *k* subsamples are concatenated. For bulk mRNAseq and ChIPseq samples, we assessed k=2 and k=3, n=10. In Section 4, we present simulated samples for k=2,3,4,5,10 for one GT sample (bulk mRNAseq, 0 h rep 1), where n=10; we also analysed simulated samples for k=2 with variable split proportions from 55-45 to 95-5. For the bulk ChIPseq data, we assessed k=2 and k=3, with n=10.

For the smartSeq data and 10× data, we generated n=3 S samples for k=2 for each of the study cases (i.e., each subset of samples), respectively, using the seqtk toolkit (v1.3-r106) (https://github.com/lh3/seqtk, accessed on 6 November 2022). We note that due to the stochasticity of sequencing, we cannot simulate fragments that are present exclusively in the whole or split samples. This limitation of the simulation study is compensated by the analysis of true split samples (the *D. melanogaster* dataset).

**[bulk mRNAseq, Yang et al. dataset]** The raw samples (no QC-based filters applied) were aligned to the *M. musculus* genome [Ensembl 98.38] [48] using STAR 2.7.6a (paired-end mode) [45]. Next, the expression was quantified using featureCounts 2.0.0 [46]. To assess the stability between S and GT samples, abundance density plots and MA plots were produced. We used noisyR [14] to perform noise analysis on the GT and S samples and further analysed the PCC distribution for binned abundances. For each dataset, expression levels were normalised using quantile normalisation [49], and DE genes between 0 h and 12 h were identified using edgeR [50]. The DE call was based on $|\log_{2}\mathrm{FC}|>0.5$ and adjusted *p*-value < 0.05 (using Benjamini–Hochberg multiple testing correction). Enrichment analysis on the resulting DE genes was performed using the gprofiler2 package [51] on GO, KEGG, reactome and transcription factor terms, with the background set as the set of expressed genes with abundance >0 in at least one sample.

**[bulk ChIPseq]** The GT and S samples were aligned to the *M. musculus* genome using bowtie2 v2.4.2 (local mode default parameters) [52]. Output SAM files were converted to BAMs, and only unique alignments were retained. Narrow peaks were called using macs2 2.2.7.1 [53]. Peaks were matched between samples if the midpoint of the peak in sample 1 is within the boundaries of the peak in sample 2 and vice versa. Across sets of samples, amplitudes were normalised using quantile normalisation [49], and differentially methylated peaks between 0 h and 12 h were identified using edgeR [50]. Peaks were called DE if $|\log_{2}\mathrm{FC}|>0.5$ and adjusted *p*-value < 0.05 (using Benjamini–Hochberg multiple testing correction).

**[sc smartSeq]** Six experimental study cases were designed based on the Cuomo dataset [42], considering the donor, time-point and cluster cell identities (Table 1). The corresponding samples were standardised in read length using Trim Galore (0.4.1) by trimming 10bp from the 5${}^{\prime }$ end (to reduce the effect of sequencing bias) and 40 bp from the 3${}^{\prime }$ end (to address high adapter sequence content); the length of the resulting reads was 75 nts. The GT and S samples were aligned to the *H. sapiens* genome (GRCh38.p13) [48] using STAR (2.7.0a) [45] in paired-end mode. The gene counts were summarised in a matrix using featureCounts 2.0.0 [46]. We applied fastQC [54] to obtain read quality metrics and multiQC [55] to aggregate QC results from the reads, alignment and quantification. Seurat objects [56] were created considering features expressed in >3 cells and cells with >50 features. The analysis pipeline comprises: (i) the normalisation of expression levels (SCTransform [57]), (ii) the computation of PCA and UMAP embeddings (RunPCA, RunUMAP), (iii) neighborhood graph computation (FindNeighbors), (iv) clustering (FindClusters), (v) and differential expression (FindAllMarkers). We considered as DE features those with $\log_{2}\mathrm{FC}$ > 0.5 (i.e., the positive markers) and an adjusted *p*-value < 0.05 (using Bonferroni multiple testing correction). To assess the similarity of the partitioning in the GT and S samples, we calculated Jaccard similarity indices (JSI, [58]) on the cluster-specific sets of DE features, restricting the JSI to the smaller set of markers.

**[sc 10× Genomics]** The GT and S fastq files were aligned to the 10× *H. sapiens* GRCh38 v3 reference transcriptome; the protein-coding genes were quantified using 10× Cellranger v3.1.0 [59]. The processing and analysis was performed individually for each donor for the GT and S samples: after the inspection of distributions of UMIs, number of detected genes, proportion of UMIs from mitochondrial genes (MT) and ribosomal protein-coding genes (RP), only cells with >1000 unique genes, <10% MT and >20% RP were retained for downstream analysis; the MT and RP genes were subsequently discarded from the count matrix. Following normalisation using SCTransform [57], the 3000 most abundant genes, accounting for 60–85% of UMIs in cells across the data, were identified and used for the calculation of PCA. A UMAP dimensionality reduction was calculated using the 30 first PCs (after the inspection of an elbow plot of PC variance); the UMAP was subsequently used to assess the extent of potential batch effects originating from the tissue origin of cells, raw and normalised sequencing depths, MT% and RP%.

A 20-nearest neighbour graph was computed on the first 30 PCs of the data; the cells were clustered using SLM community detection [60] on the NN graph. To assess the clustering similarity, element-centric clustering comparison [61] was employed on the set of common barcodes between GT and S using the ClustAssess R package [62]. Cluster markers were identified using the ROC test in Seurat v3.1.4 [56]; only genes with |FC|>2 were considered. The top 25 markers per cluster, ranked by discriminative power, were subsequently used to calculate the per-cell JSI between cluster markers; the JSI when at least one of the sets is empty was set, by default, to 0.

All analyses were performed in R (3.6.3). The code for generating these results is available on github https://github.com/Core-Bioinformatics/split-manuscript, accessed on 6 November 2022. All tests were performed on a Linux server (16 cores, 755G RAM).

## 3. Results

### 3.1. Across-Lane Split Leads to Differences in Bulk mRNAseq Data

To determine (and justify) the appropriate parameters for the simulation study, we analysed the properties of 3 *D. melanogaster* bulk mRNAseq samples, for which both the whole-sample per lane [40] and a quantification-based 50/50 split output were available. We note that the sequencing depths for the halves-samples (2RH2: 36M/26M (58%/42%), 2RA3: 21M/31M (41%/59%), 26RH3: 33M/36M (47%/53%)) diverged from the expected 50/50. To assess the consistency in properties for split samples vs. whole samples, we first evaluated the nucleotide composition; we see no significant (BH-adjusted *p*-value <0.05) differences when comparing, per base, nucleotide distributions or GC content (assessed using $\chi^{2}$ tests on A/C/G/T frequencies per position, Supplementary Table S1). Next, we looked into the number and abundance of non-redundant (unique) fragments; we illustrate the distributions of abundances for the specific fragments across the 3 available samples.

Although we see an increase in sequencing depth for the merged samples vs. the whole samples, we do not consistently observe a similar proportional increase in the number of unique reads; for 2RA3, the ratio of unique reads in the merged samples compared to the whole samples is ∼1.16 (compared to a ∼1.36 ratio on sequencing depths); we observe a similar proportion for 2RH2 (∼1.17 compared to ∼1.18) and for 26RH3 (∼1.20 compared to ∼1.23). Next, we assessed the ratio between the abundances of individual reads in the whole samples vs. the merged samples. The analysis was performed on non-zero counts for both compared samples; the ratio is expected to mirror the proportions of sequencing depths. In Supplementary Figure S1A–C, we show the distribution of $\log_{2}$ ratios of abundances between merged and whole samples binned on abundances; the guideline indicates the expected sequencing-depth-based ratio. The ratio distributions are located below the guideline for low-medium abundances (1–6 on the $\log_{2}$ scale); the ratio distributions increase to the expected level for medium-range abundances and rise above the guideline for high abundances, underlining an “over-amplification” of signal and a sensitivity of signal quantification to the lane-splitting strategy. Most specific fragments (to either the concatenated samples or the whole ones) are low-abundance; however, we note a few high-abundance fragments, which may be affected by sequencing bias (the distributions of abundances are summarised in Supplementary Figure S1D–F).

The change in the ratio between the split and whole samples is also reinforced when we consider the differences between the average abundances, calculated using all incident reads, on sliding windows (100 nts) for the whole and merged samples (Supplementary Figure S1G–I). The ratios are scaled per overall window abundance. Positive differences (red) correspond to higher amplitudes in the whole sample; negative differences (blue) correspond to higher amplitudes in the merged samples). For each sample, we show both the distribution for all differences (top subplot) and for differences above 0.2 (bottom subplot); the number of windows in each abundance bin are shown above the corresponding boxplots. We see a wider variation between the whole and merged samples at low-medium abundances (0–7 on the log${}_{2}$ scale) with larger differences when the merged-sample abundance is higher than that in the whole sample. This trend suggests a systematic, consistent stochastic over-amplification in the merged-samples; conversely, for abundances higher in the whole sample, the differences in the merged-samples are more subtle. For medium-high abundances (>7, $\log_{2}$ scale), we see a higher number of small differences for windows with abundances higher in the merged sample; the range of differences is wider for windows with higher abundance in the whole samples, but their count is lower. The pre-alignment analysis at the read level (Supplementary Figure S1A–C) hinted at these more subtle downstream effects, underlining the importance of studying the impact of splitting across lanes on real data and simulated case studies.

To illustrate some differences observed on individual transcripts, we present three examples (from sample 2RH2) of expression profiles, quantified using all the reads from the whole, the split and the merged samples for transcripts at high, medium and low abundances; these examples were selected based on the differences in signal distributions. The two split samples behave as technical replicates, as expected; however, for the merged sample, we observe significant variation in the distribution of the signal compared to the whole sample (panel L) or in the localisation of expression features such as peaks (panels J, K)). These differences may have knock-on effects on the downstream analyses and interpretation results (e.g., for the quantification of noise [14]).

These remarks on the sequenced output generate the hypothesis that conclusions drawn from standard comparisons between samples may be altered by variations in splitting strategies; this effect is also entirely technical and stems from the stochasticity of sequencing. While the simulation approach proposed in this study cannot capture fragments found exclusively in split samples, the subsampling/concatenating process mirrors the under-representation of low-medium fragments and the over-amplification of high abundance fragments, as well as loss of read diversity.

### 3.2. Consequences of Across-Lane Splitting on Bulk Data

To systematically investigate the consequences of the observed differences resulting from splitting samples across lanes, we simulated the splitting of sequencing samples on several datasets and assesses the downstream effects.

**Figure 1:**
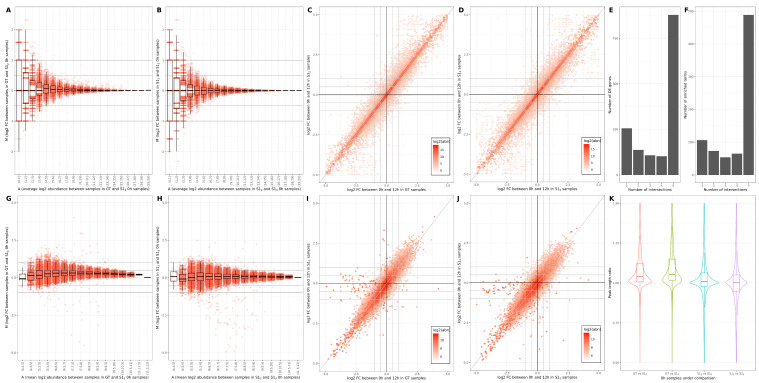
**Comparison of bulk mRNAseq analysis results for GT and S samples**. (**A**,**B**). Scatter (MA) and box plot summary on GT sample (0 h, rep1) and a corresponding k=2 S sample S1${}_{2}$ (subplot **A**) and k=2 and k=3 S samples S1${}_{2}$ and S1${}_{3}$ (subplot **B**). (**C**,**D**). Scatter (cross) plots comparing the DE amplitude ($\log_{2}\mathrm{FC}$) calculated on 0 h vs. 12 h samples for the GT vs. k=2 S samples S1${}_{2}$ (subplot **C**) and k=2 and k=3 S samples S1${}_{2}$ and S1${}_{3}$ (subplot **D**); the colour gradient is $\log_{2}$-proportional to the average abundance across all 8 corresponding samples. (**E**). Upset plot showing intersections between sets of DE genes (0 h vs. 12 h) in GT samples (GT), 2 sets of k=2 S samples (S1${}_{2}$ and S2${}_{2}$) and 2 sets of k=3 S samples (S1${}_{3}$ and S2${}_{3}$). (**F**). Upset plot showing intersections between sets of significant terms (adj *p*-value ≤0.05, BH correction) predicted using gprofiler2 on the corresponding DE genes (see (**E**)). **Comparison of H3K4me3 ChIPseq analysis results for GT and S samples.** (**G**,**H**). Scatter (MA) and box plots showing $\log_{2}$ abundance against $\log_{2}\mathrm{FC}$ within (**G**) GT sample (0 h) and a k=2 S sample S1${}_{2}$ and (**H**) k=2 and k=3 S samples S1${}_{2}$ and S1${}_{3}$ for the same sample (0 h). (**I**,**J**). Scatter (cross) plot showing $\log_{2}$FC when comparing (**I**) 0 h and 12 h for GT samples and k=2 S samples S1${}_{2}$, and (**J**) 0 h and 12 h for k=2 and k=3 S samples S1${}_{2}$ and S1${}_{3}$, coloured by the average amplitude across all 4 corresponding samples. (**K**) Violin and box plots showing distribution of ratio of peak lengths between 2 samples ($\frac{\mathrm{sample}\;1\;\mathrm{peak}\;\mathrm{length}}{\mathrm{sample}\;2\;\mathrm{peak}\;\mathrm{length}}$) comparing ground truth (GT), k=2 S sample (S1${}_{2}$ and S2${}_{2}$) and k=3 S samples (S1${}_{3}$).

**[bulk mRNAseq]** For the bulk mRNAseq study case [41], we focus on the effect of across-lane sample splitting on expression quantification, DE calling and enrichment analysis (summarised in Figure 1A–F). First, we assess the differences in quantification corresponding to the same time-point/biological replicate, 0 h (rep 1), on GT vs. k=2 S sample S${}_{2}$ (1A) and k=2 vs. k=3 samples S${}_{2}$ and S${}_{3}$ (1B). We observe standard MA funneling shapes, with a variation in excess of 1.5FC for low abundances for both comparisons. In addition, for low-to-medium abundances, we also see a wide distribution of $\log_{2}\mathrm{FC}$ in the S${}_{2}$-S${}_{3}$ comparison, underlining the technical variation (noise) that is introduced (Figure 1B). Also noted is a slight shift of FCs (Figure 1A) driven by the stochastic redistribution of reads, i.e., a lack of signal for some low-abundance genes and over-expression of a small number of medium-high abundance genes. Similar conclusions are presented in Supplementary Figure S2A,B illustrating GT vs. k=3 samples S1${}_{3}$ and two simulations of k=2 S1${}_{2}$ and S2${}_{3}$, respectively.

Next, we focus on the DE call between the 0 h vs. 12 h replicates, determined using standard pipelines and parameters. We illustrate the properties of the DE sets called on GT vs. k=2 (Figure 1C) and k=2 vs. k=3 (Figure 1D), respectively; overall, the results, converge i.e., all genes are located in the proximity of the diagonal; however, the variation is larger for the k=2 vs. k=3 simulation S1${}_{2}$ vs. S1${}_{3}$ (1D). Specifically, we see 4086 genes (14.2% of expressed genes) with a >0.5 $\log_{2}$FC absolute difference for S1${}_{2}$ vs. S1${}_{3}$ compared to 4041 (13.7%) between GT and S1${}_{2}$. The colour gradient is proportional to the average abundance of genes and underlines that medium-abundance genes are mostly affected by the variability in fold-change amplitude and identity. We also assessed the stability of the DE signal for the GT and two sets of simulations for k=2 and k=3, summarised as an upset plot (the x/5 intersections are represented, with x∈{5,4,3,2,1}). While the majority of genes are detected across all comparisons (879 out of 1092 for DE in GT and 1483 called DE in any GT or S sample), we notice comparison-specific genes (up to 79 out of 1179 called DE for S2${}_{3}$). In particular, this analysis revealed only a few “false negatives” (20 genes that were DE only in GT samples) in contrast to a larger number of “false positives” (between 42 and 79 from each S sample). A total of 18 genes (out of 1483 called DE in any GT or S sample) were identified across all S samples but not in the GT. To link back the variability of the signal to the biological interpretation of the results, we compare the sets of enriched terms corresponding to the various DE predictions (Figure 1F). Similarly to the DE summary (Figure 1E), we observe a 62.1% consistency in results, i.e., significant terms shared between all comparisons (out of 634 identified in total for GT, 787 were identified for at least one comparison). However, “false positive” entries are present (79 in total, between 13 and 27 from each S sample), identified only for one set of S samples. These corroborated results underline a mixture of consistent patterns (on S samples) and random variation that is difficult to predict or mitigate. This suggests lane-splitting introduces non-biologically-robust results.

**[bulk ChIPseq]** The effects of across-lane sample splitting are also observed on ChIPseq data. For this case study, we will focus on the changes in peak amplitude and length; H3K4me3 (Figure 1G–K and Supplementary Figure S2E–J) and H3K27ac (Supplementary Figure S2K–Q) data will be exemplified [41]. The variability on peak calling for H3K4me3 data is assessed using MA (Figure 1G,H) and cross plots (Figure 1I,J). Similarly as for the mRNAseq data, we observe a funneling behaviour when GT and S samples are compared (Figure 1G,H), a wider variation in FC for S1${}_{2}$ vs. S1${}_{3}$ (Figure 1H), and an over-amplification of peak abundances in the simulated samples (Figure 1G); additional supporting results are presented in Supplementary Figure S2E,F. These fluctuations propagate on DE calls (performed using standard pipelines on the 0 h and 12 h replicates) and are summarised in Figure 1I,J. Despite the overall convergence, for the H3K4me3 data, we notice a wider variability than for the mRNAseq data, with clear “false-negative” peaks in the simulated data, i.e., medium-abundance peaks identified as DE in the GT comparison, with a $\log_{2}\mathrm{FC}>2$ and not detected as DE in the S samples (borderline vanishing peaks); this behaviour is also observed for the H3K27ac data (Supplementary Figure S2M,N).

To further investigate the reduced robustness for the DE call, we focused on other peak properties such as peak length (defined as stop–start coordinates from the macs2 narrowPeak output); in Figure 1K, we illustrate the distribution of ratios of peak lengths when GT and S samples are compared. We notice a global, systematic shift towards shorter peaks in simulated samples; the effect is more pronounced for k=3, and a stability of behaviour is observed across simulations (blue and purple distributions). The decrease in peak length can only be observed for common peaks. The reduction in the number of called peaks is illustrated in Supplementary Figure S2I,J. For the 0 h sample (SRR7624381), we see 8910 fewer peaks called for S1${}_{2}$ samples (mean across 10 iterations) compared to GT and 11,751 fewer for S1${}_{3}$ samples compared to GT, yielding percentage decreases averaging 14.1% and 18.6%, respectively.

Simulated across-lane sampling splitting has multiple effects on the peaks called in ChIPseq data, from the number of peaks to their amplitude and length; this technical, stochastic variation may have an impact on the interpretation of the results.

### 3.3. Effects of Across-Lane Splitting on Single-Cell Data

**Figure 2:**
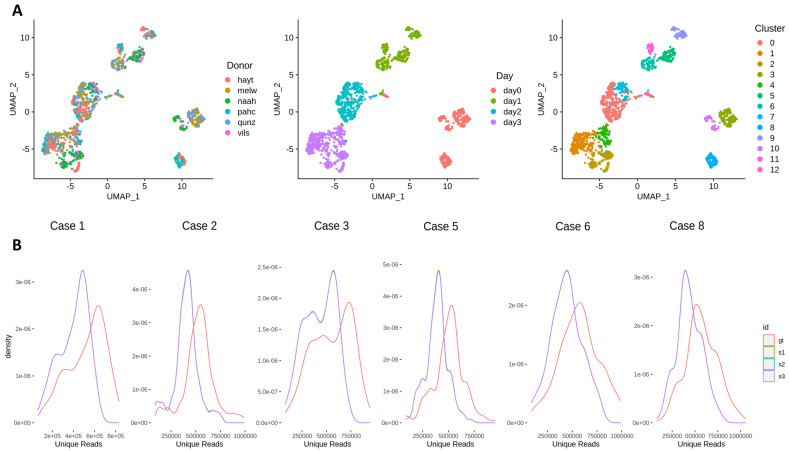

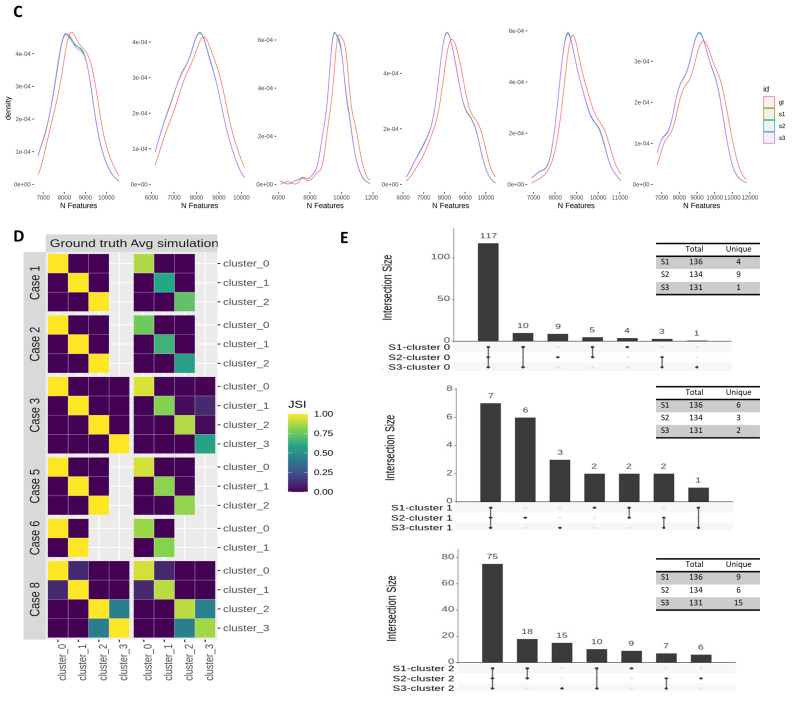
**Sample splitting across sequencing lanes introduces variability that is propagated to downstream analysis in Smart-seq2 scRNA-seq data.** (**A**) UMAP representation of the Cuomo dataset. Cells are coloured according to their donor (left), time-point (central) and cluster (right). For the different study cases considered, we show: (**B**) number of unique reads distribution for the ground truth and simulations; (**C**) number of feature distributions for the GT and S samples; (**D**) cluster similarity for each study case as evaluated using the JSI calculated on the set of DE features obtained per cluster. The values across the 3 simulations were averaged using the geometric mean. (**E**) Variability of differentially expressed feature set and number of unique DE features across simulations for cluster 0 (upper), 1 (central) and 2 (lower) for Study Case 1.

**[sc smartSeq]** We exemplify the effect of across-lane sample splitting on single-cell data first on a smartSeq2 dataset. The diversity of conditions (donors and time-points, illustrated in Figure 2A) of the Cuomo et al dataset [42] enables us to incrementally examine the consequences across 6 study cases (Table 1), covering a wide range of experimental situations.

At the sequencing read level, the sample splitting significantly reduces the number of unique reads (Figure 2B, Supplementary Table S2A), i.e., the read diversity decreases due to the expansion (over-representation) of duplicated reads. On an invariant total number of reads, this decrease propagates through to the subsequent alignment and quantification steps without influencing the fraction of mapped reads and the count distribution of samples, respectively (Supplementary Figure S3B,C); nonetheless, this results in a reduction of the number features detected in S samples (Figure 2C). This is a consequence of the loss of low-abundant features, while mid-abundance and high-abundance features slightly increase their expression due to the higher read duplication. Significant differences in the number of features (<0.01 type I error) between simulations and ground truth are detected only for study Case 3; the abundance distributions are not significantly different (in localisation or shape) across the other study cases (Supplementary Table S2A,B). Despite this, the reduction in the number of observed features has an impact on downstream analyses, such as partitioning into clusters and cell visualisation or identification of cell type markers. For the former, the UMAP topology of cells/clusters is altered (Supplementary Figure S3A); for the latter, in Figure 2D, we observe a generalised reduction in the similarity of marker genes for S samples when compared to marker genes determined on the ground truth sample (overall, 0.55–0.94 of the markers are shared between homologous clusters in GT and S samples). In addition, to assess the variability across simulations, we display summary upset plots of marker genes across clusters and simulations (Figure 2E, Supplementary Figure S3E). Although the majority of the DE entries are shared between simulations, unique features are present in most cases; these sum up to 12.3% of total features, on average, across clusters, simulations and study cases.

Overall, these results suggest that sample splitting reduces read diversity, which is propagated to downstream analyses, altering the number of features expressed in samples and potentially altering the biological interpretation of the results. Moreover, we highlight the variability across simulations, which may introduce an additional degree of irreproducibility across replicates.

**Figure 3:**
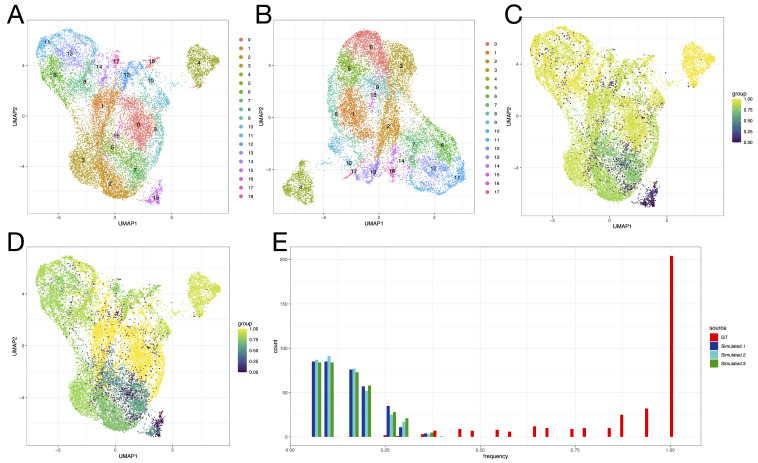
**Lane splitting induces variability in clustering of P1 10× scRNA-seq data.** (**A**,**B**): UMAP representations of GT (**A**) and S (**B**) samples, with colors indicating SLM clusters calculated on nearest-neighbor graphs; 19 clusters are found in GT and 18 in the S samples. (**C**). Element-centric clustering similarity, highlighted using the colour gradient, reveals differences between clusterings at the bottom of the UMAP, especially for the lower-right island of cells (vanishing island). (**D**). Jaccard similarity of cluster markers across GT and S clusterings suggests differences in cell types inferred from the data. (**E**). Fraction of within-island nearest neighbours for cells in the vanishing island in GT and 3 S samples. The island cells are no longer discrete from the larger body of cells in S samples.

Following SLM clustering [60] on the nearest-neighbor graph, 19 clusters were found on GT, and 18 were found on the simulation of P1 (Figure 3A,B). An element-centric clustering comparison [61] was used to evaluate the per-cell clustering similarity on the set of barcodes common to GT and simulated samples (Figure 3C), revealing high similarity (ECS>0.6) for 91% of cells (the top of the GT UMAP) and especially for the island on the upper right (cluster 4 in both clusterings); low similarity (ECS<0.2) was observed for 9.0% of cells (mainly the bottom of the UMAP), and no cells had intermediate ECS (0.2≤ECS≤0.6). Notably, an island of cells in the lower right of the GT UMAP, corresponding to cluster 15 in GT, disappeared in the split-lane simulation (S samples).

**[sc 10× Genomics]** The second single-cell case study focuses on a 10× dataset [43]; a similar strategy for generating split samples was applied. Following the alignment and protein-coding gene quantification of the GT and S samples, we observe a high overlap of called cells (i.e., barcodes) resulting from the Cellranger cell-calling algorithm (19,453 common barcodes, 166 barcodes unique to ground truth, 13 barcodes unique to the simulation). After filtering the low-quality cells, the vast majority of barcodes were still common to both versions of the analysis (17,095 barcodes in common, 155 barcodes unique to GT, 5 barcodes unique to the simulation). We observe a greater diversity of UMIs and genes in GT compared to simulated samples (Supplementary Figure S5F–G).

To investigate whether the disappearing island was a reproducible effect of the lane-splitting simulations, n=3 S samples were generated. The S samples were consistent in terms of UMAP topography and SLM clustering results (Supplementary Figure S4A–F). Across all S samples, the “island cells” were scattered in the larger body of cells (Supplementary Figure S4H). Conversely, in UMAPs generated on GT across 4 random seeds, the island of cells was consistently detached from the larger body of cells (Supplementary Figure S4G). Furthermore, the fraction of within-island nearest neighbors for the island cells was quantified in the GT and S samples (Figure 3E); while the island cells had each other as neighbors in GT, this was no longer observed in the simulations, suggesting the loss of some transcriptional heterogeneity during the lane splitting.

The marker genes that distinguish the vanishing island (Supplementary Figure S5A) from the rest of the cells are SPP1, SRGN, SOCS2, ALDH1A1, AREG and HIST1H1C (Supplementary Figure S5B); in particular, SPP1 appears highly specific to the island. Upon recalculating the PCA, and subsequently the UMAP, on the set of abundant genes with SPP1 excluded, the island is absorbed into the wider body of cells (Supplementary Figure S5C), and so is SPP1 expression (Supplementary Figure S5D). SPP1 is identified as the 5th most variable gene in GT by SCTransform. In S samples, marked differences in gene variance can be observed when compared to GT (Supplementary Figure S5E), leading to the downstream consequences on dimensionality reductions and clustering results.

To further investigate the consequences of lane-splitting clustering variability on cluster markers, typically used to infer the identity of cells, the per-cell Jaccard similarity index (JSI) was calculated (Figure 3D). Certain regions of cells, such as the middle-right of the UMAP, displayed high JSI, indicating they would be interpreted similarly in the GT and S analyses. Other regions exhibited lower JSI; the vanishing island (cluster 15) had a lower JSI than the retained island (cluster 4). These results suggest that the former could be interpreted inconsistently, depending on whether the library was split across lanes or not.

## 4. Discussion

### 4.1. Effects of Splitting on Read Diversity and Levels of Noise

**Figure 4:**
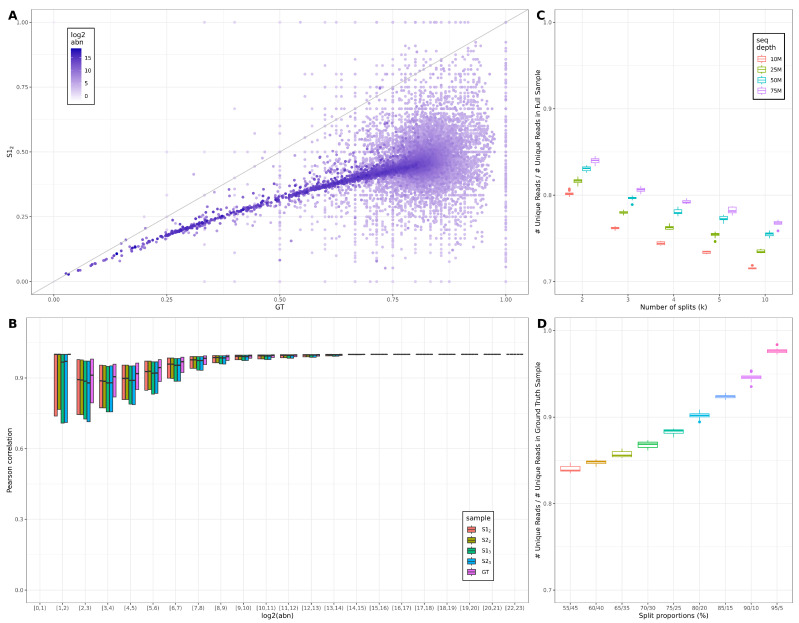
**Summary of split effects on the number of unique reads and noise.** (**A**) Scatter plot illustrating the complexity ratio (non-redundant to redundant counts) for the GT (x-axis) vs. an S (y-axis) sample for SRR7624365 (bulk mRNAseq, 0 h rep 1). Each point represents a gene, and the colour gradient is proportional to the $\log_{2}$ abundance. (**B**) Box plot of the PCC binned by abundance for the transcript-based noise removal (noisyR applied to BAM files) corresponding to GT, k=2 and k=3 S samples for 0 h rep 1. (**C**) Boxplot showing distributions of ratios of recovered unique reads in the S samples with respect to the *k* hyper-parameter (k=2,3,4,5,10); the effect of GT sample sequencing depth is also assessed. The distributions are built on 10 iterations. (**D**) Boxplot showing distribution of ratios of recovered unique reads in the S samples when the proportions of concatenated subsamples are varied (from 55-45 to 95-5). The distributions are built on 10 iterations.

The main consequence of across-lane sample splitting is the variation in read diversity; this propagates onto transcript coverage and quantification. To assess the variation, we focused on the transcript complexity (defined as the ratio of unique to total reads [63], calculated per transcript), exemplified on the SRR7624365 sample (bulk mRNAseq, 0 h rep 1). We compared the complexities, per transcript, of the GT and S samples (Figure 4A); each point corresponds to a gene, and the colour gradient is proportional to the $\log_{2}\left(\mathrm{abn}\right)$. We observe a consistent trend for medium-high abundant genes across a wide complexity range [0,0.75], highlighting the higher complexity (i.e., more diverse reads) of the GT sample. Also noticeable is the high variability in complexity for the low-abundance genes, presented on the MA plots in the bulk section of the Results, and the localisation of the low-abundance cloud under the equal complexity diagonal, enforcing the previous conclusion across all abundances.

Yet another side effect of the variation in reads diversity is the quantification of noise across the samples [14]; we focused on the transcript-based approach in noisyR since it is directly influenced by the robustness of transcript coverage. In Figure 4B, we illustrate the variation of point-to-point PCC vs. the variation in abundance for the GT and 2 iterations of k=2 (S1${}_{2}$,S2${}_{2}$) and k=3 (S1${}_{3}$,S2${}_{3}$). The wide and low PCC distributions correspond to higher levels of noise; the distributions become higher and tighter for medium- to high-abundance genes. For medium-abundance genes, the GT distributions are systematically higher than k=2 and k=3, suggesting that the splitting increases the level of noise and thus interferes with the detection of DE genes or other downstream analyses, as illustrated in the Section 3. The level of noise in the simulated samples is also variable and generally higher in k=3 samples than k=2, although the distribution of PCC is wider and lower for all S samples than GT.

### 4.2. Effects of Varying the Number and Proportions of Splits

In the Section 3, we exemplified the concatenation of k=2 and k=3 equal subsamples. However, in a real-world scenario, we rarely observe an equal number of reads across split samples; in addition, split designs are occasionally mixed. To further understand the knock-on effects on downstream analyses, we illustrate the effect of the variable number and proportions of reads allocated to splits. We used the bulk mRNAseq sample SRR7624365 (0 h rep 1) as the starting point, and, based on the consistency in conclusions across different inputs, we expect a convergence of results for other bulk or single-cell data. We subsampled the fastq files to 50%, 33%, 25%, 20% and 10% of the total sequencing depth and created S samples by concatenating the 2, 3, 4, 5 and 10 subsamples, respectively. For each case study, the distributions were generated on 10 iterations. We also assessed the sequencing depth co-variate; the results on the full sequencing depth (75.4 M) and for samples subsampled to 50 M, 25 M and 10 M reads, respectively, (without replacement) are presented. The ratios of recovered unique reads (i.e., number of unique reads in the S sample divided by the number of unique reads for the GT sample) across the simulated case studies are presented in Figure 4C. We observe a decrease in recovered ratios proportional to the number of splits and initial sequencing depth. The decrease in ratios is uniform across sequencing depths, but the recovered ratios also have lower y-intercepts (lower absolute ratios), highlighting that the effect of across lane-splitting is more extreme at low sequencing depths, i.e., while the decrease-rate is lower for higher *k*-values, as the number of splits (*k*) increases, we see a consistent reduction in the number of unique reads. This underlines that splitting across lanes has an adverse effect on the diversity of reads.

Additionally, we assessed the effect of uneven splits across lanes; focusing on k=2 S samples, we varied the split proportions from 55-45 to 90-10. The resulting recovery ratios are shown in Figure 4D. The minimum for the recovery ratios is achieved for the 50-50 proportions; the recovery ratios gradually increase as the larger subsample approaches 100%. The observed increase is small for the first few increments and increases more rapidly as proportions approach 100-0. This illustrates, from yet another angle, the variation in read diversity.

## 5. Conclusions

On various sequencing datasets, bulk and single-cell, we observed that the splitting of samples across lanes reduces the diversity of reads, which in turn triggers side effects on quantification (e.g., gene expression for mRNAseq, peak expression for ChIPseq) and auxiliary properties (such as the length of ChIP peaks). The splitting in itself introduces an additional level of variability in terms of robustness and reproducibility; it may pose added difficulties stemming from the variable observed number of reads derived from the stochasticity of the sequencing itself. The potential batch effects derived from loading full samples on sequencing lanes can be mitigated through randomisation. We acknowledge that technical circumstances may make splitting unavoidable; our recommendation is consistency in sequencing setup across all samples in an experiment.

## Figures and Tables

**Table 1:** Overview of the study cases built on scRNA-seq Smart-seq data (Cuomo et al., 2020, [42]) illustrating the combinations of the different covariates. Study case 4 (same donor, different time-point and same cluster) and case 7 (different donor, different time-point, and same cluster) could not be generated due to the data structure. While an insufficient number of cells fell into these two study cases (4 and 7) in this instance, they do represent valid study cases that should be considered for similar designs and are therefore still included.

Study Case	N Cells	Donor	N Cells (Donor)	Time Point	N Cells (Time Point)	Cluster	N Cells (Cluster)
Case 1	105	hayt	105	day2	105	0	105
Case 2	106	pahc	106	day3	106	1	66
						4	40
Case 3	168	melw	94	day0	168	3	168
		qunz	74				
Case 5	168	hayt	168	day1	61	9	61
				day3	107	1	107
Case 6	95	melw	47	day1	95	5	45
		vils	48			6	50
Case 8	217	melw	95	day0	95	3	95
		naah	122	day3	122	2	122

## Data Availability

All code and processed data presented in this study are available on the Core Bioinformatics github page: https://github.com/Core-Bioinformatics/split-manuscript, accessed on 6 November 2022.

## Supplementary Materials

The following supporting information can be downloaded at: https://www.mdpi.com/article/10.3390/genes13122265/s1
